# Dietary Arginine Supplementation Modulates Nitrogen Utilization, Serum Biochemistry, and Gut Microbiota in Growing Beagles Under Low- and Normal-Protein Diets

**DOI:** 10.3390/ani16152385

**Published:** 2026-08-03

**Authors:** Mengdi Zhao, Yueyao Li, Yuanyuan Zhang, Yixin Wang, Xiaorui Zhang, Shuang Liang, Xinkang Li, Guangyu Li

**Affiliations:** College of Animal Science and Technology, Qingdao Agricultural University, Qingdao 266109, China; zmdi0807@126.com (M.Z.); 20222203016@stu.qau.edu.cn (Y.L.); 20222103021@stu.qau.edu.cn (Y.Z.); 20242209016@stu.qau.edu.cn (Y.W.); 20242109049@stu.qau.edu.cn (X.Z.); 20232209008@stu.qau.edu.cn (S.L.); 20232209006@stu.qau.edu.cn (X.L.)

**Keywords:** low-protein diet, arginine, beagles, growth performance, metabolomics

## Abstract

This study investigated the effects of dietary protein levels and arginine supplementation on growth performance, nitrogen metabolism, serum biochemical indices, inflammatory markers, gut microbiota, and fecal metabolomic profiles in growing beagles. Sixty healthy beagles were assigned to six dietary treatments in a 2 × 3 factorial design: two crude protein levels (normal, 22%; low, 18%) and three arginine supplementation levels (0%, 0.266%, 0.532%) over 45 days. Growth performance and nutrient digestibility were not significantly affected by diet. Low-protein diets reduced nitrogen intake and excretion while increasing biological value, indicating improved nitrogen utilization. Serum total cholesterol, blood urea nitrogen, and creatinine were influenced by protein and arginine levels, and inflammatory markers TNF-α and IL-6 were modulated by their interaction. Gut microbiota composition was altered at the taxonomic level but not at the level of the overall structure; arginine supplementation increased microbial richness under normal protein conditions. Fecal metabolomic analysis showed that arginine mainly affected amino acid metabolism, mineral absorption, and bile acid pathways, whereas protein reduction impacted lipid metabolism, bile secretion, and energy-related pathways. Overall, reducing dietary protein to 18% improved nitrogen utilization without impairing growth. Among the six dietary treatments, the normal-protein diet supplemented with moderate arginine (22% CP + 0.266% L-arginine, 1.10% total arginine) was associated with the most balanced overall physiological response. Metabolomic findings from the four selected dietary groups provided additional evidence that dietary protein and arginine modulated distinct metabolic pathways, although these findings should be interpreted within the scope of the selected-group comparison. These results provide a practical basis for optimizing amino acid balance and nitrogen utilization in growing dogs and may contribute to the development of more precise and sustainable pet food formulations.

## Simple Summary

This study evaluated how different protein levels and added arginine affect growth, metabolism, and gut health in growing beagle dogs. Sixty beagles were fed diets with either normal or reduced protein levels, combined with different amounts of arginine supplementation. The results showed that lowering dietary protein did not affect growth but improved nitrogen utilization efficiency, while total nitrogen retention remained unchanged due to reduced nitrogen intake. Arginine supplementation influenced blood health markers, inflammation, gut bacteria, and metabolic pathways in the feces. Overall, a moderate amount of arginine added to a normal-protein diet produced the most balanced health and metabolic benefits. These findings suggest that carefully adjusting protein and amino acid levels can improve metabolic health and support more sustainable dog food formulations.

## 1. Introduction

Recently, the global pet population has grown steadily, and pets are increasingly regarded as integral family members. This shift has promoted the development of pet nutrition from basic dietary provision toward precision nutrition and long-term health management [1]. At the same time, the expansion of the pet food industry has increased the demand for high-quality protein ingredients. Conventional high-protein pet diets often rely heavily on animal-derived protein sources, the production of which is associated with considerable resource consumption and environmental burdens, posing challenges to the sustainable development of the pet food industry [2]. Therefore, reducing dietary crude protein (CP) levels while maintaining an appropriate amino acid profile has become a potential strategy for improving protein utilization efficiency and reducing resource pressure. In this context, low-protein diets fortified with functional amino acids, such as arginine, may provide a promising approach to balancing nutritional adequacy and sustainability in pet food production [3].

Arginine, commonly considered an essential or conditionally essential amino acid in dogs, plays an important role in canine nutrition and metabolism. It is not only a key component of protein synthesis but also participates in multiple physiological processes, including nitric oxide synthesis, ammonia detoxification, hormone secretion, and immune regulation [4]. Growing dogs have relatively high amino acid requirements due to rapid growth and active metabolism, and endogenous arginine synthesis may be insufficient to fully meet physiological demands, making dietary supply particularly important [5]. Most studies on arginine requirements focus on normal dietary protein levels. Others have targeted specific physiological conditions, such as pregnancy and disease [6,7]. However, applying these findings to healthy growing dogs requires caution, as they have higher demands for both the quantity and balance of amino acids [8,9]. Under low-protein conditions, the reduction in total protein may change the amino acid supply pattern [10]. This may make amino acid imbalance more pronounced and may affect growth performance, nitrogen utilization efficiency, and metabolic health [11]. Under low-protein conditions, arginine supplementation may help improve amino acid utilization efficiency and alleviate the adverse effects associated with reduced dietary protein levels, including impaired growth performance, decreased protein synthesis, disrupted nitrogen metabolism, and compromised physiological functions resulting from insufficient amino acid availability [12,13]. However, Gessert et al. [14] suggested that single-amino-acid supplementation in low-protein diets for puppies should be used with caution, because supplementation of one amino acid alone may not overcome potential limitations in other amino acids and may disrupt amino acid balance. Nevertheless, targeted supplementation of specific functional amino acids, such as arginine, may provide metabolic benefits when the dietary amino acid profile is carefully balanced. Therefore, further investigation is needed to determine whether arginine supplementation can improve physiological responses under reduced-protein conditions in growing dogs.

It remains unclear whether arginine supplementation can effectively improve amino acid balance and protein utilization in low-protein diets and whether its metabolic effects differ under different dietary protein levels. We hypothesized that arginine supplementation exerts differential physiological effects depending on dietary protein availability, with its metabolic responses differing between normal- and low-protein conditions. In contrast, under low-protein conditions, arginine may serve as a functional limiting amino acid and exert broader regulatory effects through pathways related to nitric oxide synthesis, metabolic signaling, and immune modulation. Therefore, the present study aimed to evaluate the effects of arginine supplementation under normal-protein and low-protein dietary conditions on growth performance, nutrient digestibility, nitrogen metabolism, serum biochemical indices, and gut microbiota in growing beagles.

## 2. Materials and Methods

### 2.1. Experimental Diets

Dietary formulations were designed in accordance with the Nutrient Requirements of Dogs and Cats [15]. The diets were arranged in a 2 × 3 factorial design, with two CP levels and three graded levels of supplemental L-arginine. The two protein levels were designed as normal-protein diets (NP, approximately 22% CP) and low-protein diets (LP, approximately 18% CP). The L-arginine supplementation levels were selected based on the arginine nutritional requirements of growing dogs and previous dietary arginine supplementation studies. Specifically, supplementation with 0.266% and 0.532% L-arginine increased the total dietary arginine concentrations to approximately 1.10% and 1.35%, respectively, representing moderate and high arginine supplementation levels. These doses were selected to evaluate the dose-dependent effects of arginine under different dietary protein conditions while avoiding excessive amino acid supplementation. Within each protein level, L-arginine was supplemented at 0%, 0.266%, or 0.532%.

Crystalline L-arginine (≥98.5%, XJ Bio, Shenyang, China) was used as the supplemental arginine source. To maintain comparable dietary energy and nutrient balance, arginine was incorporated into the diets before extrusion by replacing an equivalent amount of cooked tapioca flour. All ingredients were weighed, ground to pass through a sieve, thoroughly mixed, and processed into extruded diets using an industrial extruder. After extrusion, the diets were cooled to room temperature, packed in sealed bags, and stored in a dry and ventilated room until feeding.

The six dietary treatments were designated as follows: NPI, NP diet without supplemental L-arginine; NPII, NP diet with 0.266% L-arginine; NPIII, NP diet with 0.532% L-arginine; LPI, LP diet without supplemental L-arginine; LPII, LP diet with 0.266% L-arginine; and LPIII, LP diet with 0.532% L-arginine. The detailed ingredient composition and nutrient levels of the experimental diets are shown in Table 1, while the diet composition on a dry matter basis and the amino acid composition of the main raw materials are provided in Tables S1 and S2, respectively.

### 2.2. Experimental Design

**Figure 1:**
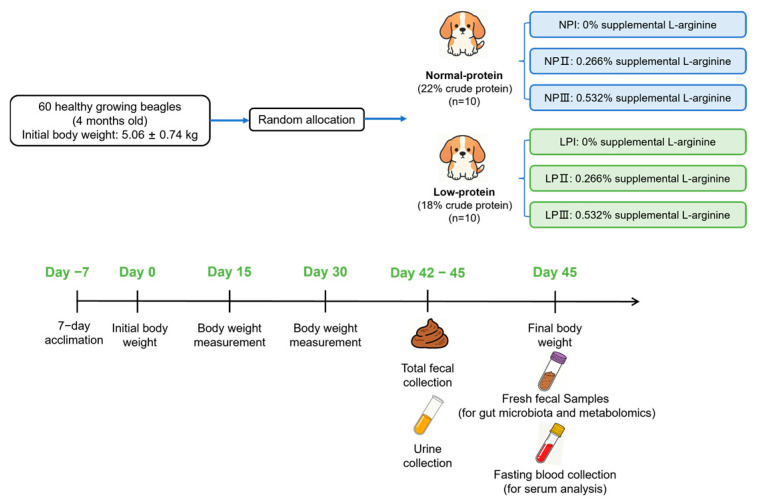
Study design.

The 60 healthy growing beagles used in this study were 4 months old and consisted of an equal number of males and females (30 males and 30 females). Dogs were randomly assigned to one of six dietary treatments, with 10 dogs per treatment (5 males and 5 females). Dogs were housed individually in separate pens (4.8 × 7.6 m) and acclimated for 7 days prior to the commencement of the 45-day experimental trial (Figure 1). Each dog was considered the experimental unit. Feed portions were calculated based on individual body weight and energy requirements and provided in two equal meals at 09:00 and 16:00 daily. Water was available ad libitum. Health status, general behavior, and feed intake were monitored daily. Body weight was measured on days 0, 15, 30, and 45, and feed amounts were adjusted accordingly. All dogs were vaccinated against rabies and regularly dewormed. The experiment was conducted at Qingdao Bolong Experimental Animal Co., Ltd. (Qingdao, China). The animals were accommodated in a room with the temperature set at 22.0 ± 2.0 °C and a 12 h/12 h light-dark cycle.

### 2.3. Sample Collection and Analysis

Representative samples of each diet were analyzed in triplicate for dry matter, CP, ether extract, ash, calcium, phosphorus, and amino acid composition. Amino acid concentrations, except tryptophan, were determined after hydrolysis with 6 mol/L HCl at 110 °C using an automatic amino acid analyzer (Hitachi L-8800, Hitachi High-Tech Corporation, Tokyo, Japan). Dry matter contents were determined according to AOAC (934.01) methods; CP levels were determined according to the Kjeldahl method, and CP was calculated as nitrogen × 6.25. Ether extract was measured using Soxhlet extraction.

Apparent total tract digestibility was determined using the total fecal collection method during the final four days of the 45-day feeding trial. All fresh fecal samples were collected and stored at −20 °C. After the trial, fecal samples from each dog were thawed, pooled, and mixed. Approximately 300 g was weighed out, dried to constant weight at 65 °C, ground to pass through a 40-mesh sieve, and kept at −20 °C for nutrient analysis. During the same collection period (days 42 to 45), urine samples were collected daily using metabolic cages. The urine collection containers were supplemented with 10% H_2_SO_4_ to prevent nitrogen loss caused by ammonia volatilization during collection. The total urine volume from each dog was measured using a graduated cylinder and recorded daily. After removing visible debris by filtration, urine samples were pooled for each dog throughout the collection period, and stored at −20 °C until analysis. Urinary nitrogen concentration was determined using the Kjeldahl method.

Apparent total tract digestibility was calculated as follows:

$$\mathrm{Dry}\;\mathrm{matter}\;(\mathrm{DM})\;\mathrm{digestibility}\;(\%)=\frac{\mathrm{DM}\;\mathrm{intake}-\mathrm{fecal}\;\mathrm{DM}\;\mathrm{output}}{\mathrm{DM}\;\mathrm{intake}}\times 100$$

$$\mathrm{Nutrient}\;\mathrm{digestibility}\;(\%)=\frac{\mathrm{Nutrient}\;\mathrm{intake}-\mathrm{fecal}\;\mathrm{nutrient}\;\mathrm{output}}{\mathrm{Nutrient}\;\mathrm{intake}}\times 100$$

Nitrogen metabolism indices were calculated as follows:


N retained = N intake − fecal N output − urinary N output

$$\mathrm{Net}\;\mathrm{protein}\;\mathrm{utilization}\;(\%)=\frac{N\;\mathrm{retained}}{N\;\mathrm{intake}}\times 100$$

$$\mathrm{Biological}\;\mathrm{value}\;(\%)=\frac{N\;\mathrm{retained}}{N\;\mathrm{intake}\;-\;\mathrm{fecal}\;N}\times 100$$

For microbial and metabolomic analyses, fresh fecal samples (5–6 g) were collected from each dog on day 45. The samples were immediately frozen in liquid nitrogen and stored at −80 °C. On day 45, blood samples were collected from the forelimb vein before morning feeding after an overnight fast. Serum was obtained by centrifugation at 2015× *g* for 15 min and stored at −80 °C until analysis.

### 2.4. Serum Biochemical Analysis

A series of serum biochemical parameters was quantified using commercial kits (Nanjing Jiancheng Bioengineering Institute, Nanjing, China). This panel included markers of liver and kidney function, including total protein (TP), alanine aminotransferase (ALT), aspartate aminotransferase (AST), creatinine (CRE), and blood urea nitrogen (BUN), as well as lipid metabolism indicators, including total cholesterol (T-CHO) and triglycerides (TG). Concurrently, oxidative stress status was assessed by measuring superoxide dismutase (SOD), malondialdehyde (MDA), and glutathione (GSH) levels with kits (Gresix Biotechnology, Beijing, China). Furthermore, immune and inflammatory markers, comprising immunoglobulin G (IgG), secretory immunoglobulin A (sIgA), tumor necrosis factor-α (TNF-α), and interleukin-6 (IL-6), were determined via ELISA kits (Jiangsu Meimian, Yancheng, China). All procedures adhered to the manufacturers’ instructions, and absorbance was read on a microplate reader.

### 2.5. Gut Microbiota Analysis

Fecal microbial DNA was extracted from frozen fecal samples using a commercial DNA extraction kit. The V_3_–V_4_ region of the bacterial 16S rRNA gene was amplified using universal primers 338F and 806R. PCR amplification was performed using Phusion High-Fidelity PCR Master Mix (New England Biolabs, Ipswich, MA, USA). The amplified products were purified using magnetic beads, quantified using a Qubit fluorometer (Thermo Fisher Scientific, Waltham, MA, USA), and pooled at equimolar concentrations for library construction. Sequencing libraries were prepared using standard Illumina library preparation reagents and sequenced on an Illumina MiSeq platform (Illumina Inc., San Diego, CA, USA) using paired-end sequencing chemistry. Raw sequencing reads were demultiplexed, quality-filtered, merged, and processed using QIIME2 software (version 2024.2). Quality control was performed using fastp (version 0.23.1), paired-end reads were merged using FLASH (version 1.2.11), and chimeric sequences were removed using the DADA2 pipeline implemented in QIIME2. Bioinformatics and statistical analyses were performed using QIIME2 and R software (version 4.0.3), with statistical analyses and visualization conducted using R packages including vegan and ggplot2. Alpha diversity indices were compared using the Wilcoxon rank-sum test. LEfSe analysis was performed using an LDA score threshold of >4.0 and *p* < 0.05. Multiple comparisons were adjusted using the Benjamini–Hochberg false discovery rate (FDR) correction.

### 2.6. Non-Targeted Metabolomic Analysis

Following thawing at 4 °C, fecal metabolites were extracted with a pre-cooled solvent mixture of methanol, acetonitrile, and water (2:2:1, *v*/*v*/*v*). The extraction procedure involved vortexing, followed by 30 min of low-temperature sonication, a 10 min incubation at −20 °C, and centrifugation at 14,000× *g* for 20 min (4 °C). The resulting supernatant was then subjected to vacuum drying. The dried residue was reconstituted in 100 µL of a 1:1 (*v*/*v*) acetonitrile/water solution, vortexed again, and centrifuged prior to analysis. We performed metabolic profiling using UHPLC-Q-TOF MS (Agilent Technologies, Santa Clara, CA, USA), acquiring data in both positive and negative ionization modes. Raw MS data were converted to mzXML format using ProteoWizard (version 3.0) and processed using XCMS (version 4.0) for peak detection, alignment, retention time correction, and peak area extraction. Metabolite identification and subsequent statistical analyses were performed based on the processed data. Differential metabolites were selected based on multivariate and univariate statistical analyses. Metabolites with variable importance in projection (VIP) values > 1.0 from the OPLS-DA model, fold change (FC) >1.5 or <0.67, and FDR-adjusted *p* < 0.05 were considered significantly different metabolites.

### 2.7. Statistical Analysis

Statistical analyses were performed using the general linear model (GLM) procedure in SAS 9.2 (SAS Institute Inc., Cary, NC, USA), with protein level and arginine supplementation as fixed factors, including their interaction. Two-way ANOVA was applied to evaluate main and interaction effects. *p* < 0.05 was considered statistically significant, while *p* > 0.05 was considered to indicate no significant difference.

## 3. Results

### 3.1. Feed Intake and Growth Performance

Throughout the experimental period, all dogs remained clinically healthy, with no abnormal signs observed. As shown in Table 2, no significant differences were found among the six dietary groups in initial body weight, 15-day body weight, 30-day body weight, final body weight, average daily feed intake, or average daily gain (*p* > 0.05). Two-way ANOVA further indicated that neither dietary CP level, arginine supplementation, nor their interaction significantly affected any growth performance parameter or feed intake (*p* > 0.05). Overall, these results suggest that reducing dietary crude protein from 22% to 18% and supplementing arginine at the tested levels did not significantly affect growth performance in growing beagles.

### 3.2. Nutrient Apparent Digestibility and Nitrogen Metabolism

The apparent nutrient digestibility and nitrogen metabolism parameters are presented in Table 3. Two-way ANOVA showed that dietary CP level significantly affected dry matter (DM) digestibility (*p* = 0.032), nitrogen intake (NI, *p* < 0.001), urinary nitrogen (UN, *p* < 0.001), and biological value (BV, *p* = 0.021). Specifically, DM digestibility was significantly lower in the 22% CP groups than in the 18% CP groups (*p* = 0.032), whereas NI and UN were significantly higher in the 22% CP groups (*p* < 0.001). In addition, BV was significantly lower in the 22% CP groups compared with the 18% CP groups (*p* = 0.021). CP level had no significant effects on the digestibility of CP, ether extract (EE), ash, calcium (Ca), or phosphorus (P), nor on fecal nitrogen (FN), urinary nitrogen excretion ratio (UR), or net protein utilization (NPU) (*p* > 0.05). Arginine supplementation did not exert significant main effects on any of the measured parameters (*p* > 0.05), and no significant CP × Arg interactions were observed (*p* > 0.05).

### 3.3. Serum Biochemical Parameters

According to the results of the two-way ANOVA, dietary CP level had highly significant effects on serum T-CHO, BUN, and CRE concentrations (*p* < 0.001; Table 4), but had no significant effects on TG, TP, ALT, or AST (*p* > 0.05). Arginine supplementation similarly exerted significant effects on T-CHO, BUN, and CRE (*p* < 0.001), whereas no significant effects were observed on TG, TP, ALT, or AST (*p* > 0.05). In addition, significant CP × Arg interactions were detected for TG (*p* = 0.040), BUN (*p* = 0.004), and CRE (*p* = 0.048), while no significant interactions were found for T-CHO, TP, ALT, or AST (*p* > 0.05).

Specifically, within the 22% CP group, arginine supplementation significantly reduced serum T-CHO, BUN, and CRE concentrations. In the 18% CP group, the 0.532% Arg treatment showed overall lower levels of T-CHO, BUN, and CRE. Compared with the 22% CP groups, the 18% CP treatments generally decreased T-CHO and BUN concentrations, whereas CRE levels were more evidently influenced by the interaction between CP and Arg. TG exhibited only a significant CP × Arg interaction, suggesting that the effect of arginine on TG varied depending on protein level. No significant differences were observed in TP, ALT, or AST among treatments, indicating that reducing dietary crude protein and supplementing arginine did not exert adverse effects on serum total protein or liver function-related indices.

According to the results of the two-way ANOVA, dietary CP level significantly affected serum IgG (*p* = 0.029; Table 5), IL-6 (*p* < 0.001), and MDA (*p* = 0.011) concentrations, but had no significant effects on sIgA, TNF-α, SOD, or GSH (*p* > 0.05). Arginine supplementation showed no significant main effects on any of the serum immune or antioxidant parameters (*p* > 0.05). In addition, significant CP × Arg interactions were observed for IgG (*p* = 0.034), TNF-α (*p* = 0.001), and IL-6 (*p* = 0.025), whereas no significant interactions were detected for sIgA, SOD, MDA, or GSH (*p* > 0.05).

Specifically, within the 22% CP group, the 0.532% Arg treatment exhibited significantly higher IgG levels compared with the 0% Arg group. In contrast, within the 18% CP group, the 0% Arg group showed relatively higher IgG levels, while the 0.266% Arg group was significantly lower. For TNF-α, although neither CP nor Arg alone had a significant main effect (*p* > 0.05), a significant CP × Arg interaction was detected (*p* = 0.001). TNF-α levels showed a decreasing trend with increasing Arg supplementation within both CP groups, but the magnitude of reduction differed between protein levels. IL-6 concentrations were significantly affected by CP level (*p* < 0.001) and showed a significant CP × Arg interaction (*p* = 0.025). Within the 18% CP group, the 0.532% Arg treatment significantly reduced IL-6 levels compared with the 0% Arg group (*p* < 0.05). In contrast, no significant differences among Arg treatments were observed within the 22% CP group. MDA was significantly affected by CP level, with overall higher values observed in the 18% CP group than in the 22% CP group, indicating that low-protein diets may influence lipid peroxidation. No significant differences were observed in sIgA, SOD, or GSH among treatments, suggesting that reducing dietary crude protein and supplementing arginine had limited effects on certain mucosal immune and antioxidant parameters.

### 3.4. Gut Microbiota Composition

**Figure 2:**
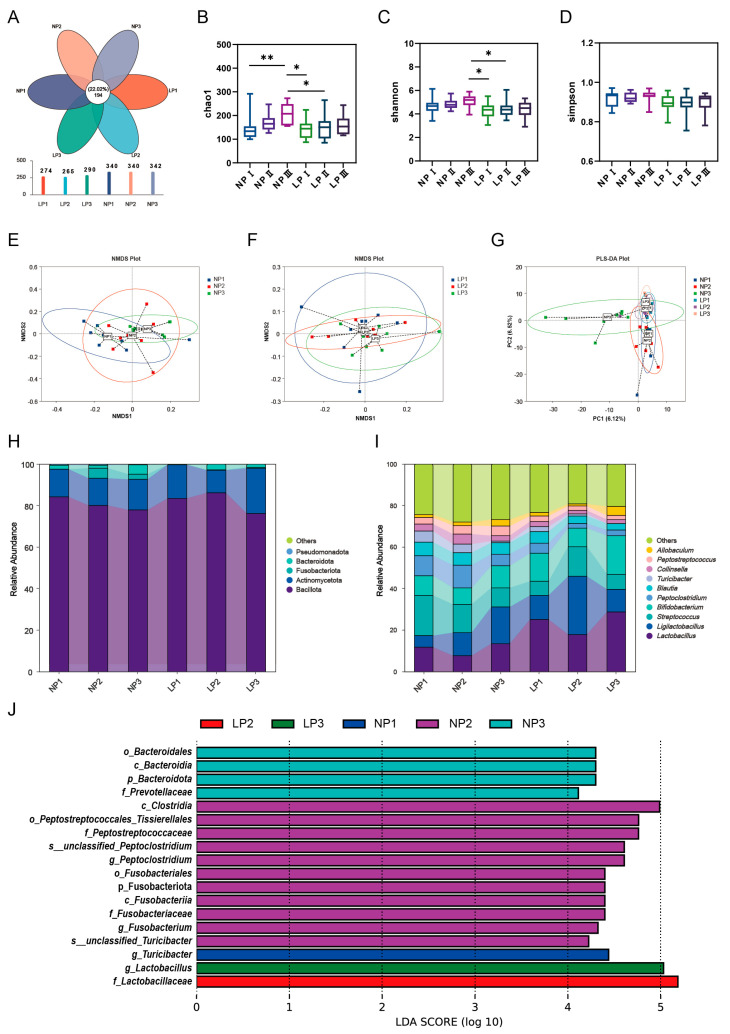
Effects of dietary arginine supplementation on the intestinal microbiota of growing beagles fed diets with different crude protein levels. (**A**), ASV flower diagram showing shared and unique ASVs among groups; (**B**–**D**), alpha diversity indices, including Chao1, Shannon, and Simpson indices; (**E**,**F**), NMDS analysis of intestinal microbial communities in the NP and LP groups, respectively; (**G**), PLS-DA analysis of intestinal microbial community structure among groups; (**H**), relative abundance of intestinal microbiota at the phylum level; (**I**), relative abundance of intestinal microbiota at the genus level; (**J**), LEfSe analysis showing significantly enriched bacterial taxa among groups with an LDA score > 4.0. NP, normal-protein diet; LP, low-protein diet; I, II, and III indicate different arginine supplementation levels. * indicates significant differences among groups (*p* < 0.05), and ** indicates highly significant differences among groups (*p* < 0.01).

The number of amplicon sequence variants (ASVs) was lower in the LP groups than in the NP groups, whereas ASV richness tended to increase with increasing arginine supplementation within the LP and NP diets (Figure 2A). Alpha diversity analysis showed that the Chao1 index was significantly higher in the NPIII group than in the NPI, LPI, and LPII groups (*p* < 0.05; Figure 2B). The Shannon index was also significantly higher in the NPIII group than in the LPI and LPII groups (*p* < 0.05; Figure 2C), whereas no significant differences were observed in the Simpson index among groups (*p* > 0.05; Figure 2D).

Beta diversity analysis based on non-metric multidimensional scaling (NMDS) and partial least-squares discriminant analysis (PLS-DA) did not show clear separation among dietary groups (Figure 2E–G), suggesting that dietary protein level and arginine supplementation mainly affected specific bacterial taxa rather than causing a marked shift in the overall microbial community structure. At the phylum level, Bacillota, Actinomycetota, Fusobacteriota, and Bacteroidota were the dominant bacterial phyla across all groups (Figure 2H). At the genus level, *Lactobacillus*, *Ligilactobacillus*, *Streptococcus*, and *Bifidobacterium* were the predominant genera (Figure 2I).

Differential taxonomic analysis showed that *Lactobacillaceae* was enriched in the LPII group, whereas *Lactobacillus* was enriched in the LPIII group. In the NPII group, the representative taxa included Clostridia, Peptostreptococcales-Tissierellales, Peptostreptococcaceae, Peptoclostridium, Fusobacteriota, Fusobacteriales, Fusobacteriia, Fusobacteriaceae, and Fusobacterium. In the NPIII group, Bacteroidales, Bacteroidia, Bacteroidota, and Prevotellaceae were identified as the main differential taxa. These results suggest that the microbial response to arginine supplementation differed according to dietary protein level.

### 3.5. Fecal Metabolomics Analysis in Selected Representative Groups

Untargeted metabolomic profiling was performed in four representative dietary groups as an exploratory analysis to investigate metabolic responses associated with dietary protein reduction and arginine supplementation. Based on the experimental design, the NPI group was used as the reference group, whereas NPII and NPIII were selected to evaluate the effects of different arginine supplementation levels under the normal-protein diet, and LPI was selected to evaluate the effect of reducing dietary crude protein without additional arginine supplementation.

#### 3.5.1. Principal Component Analysis (PCA)

**Figure 3:**
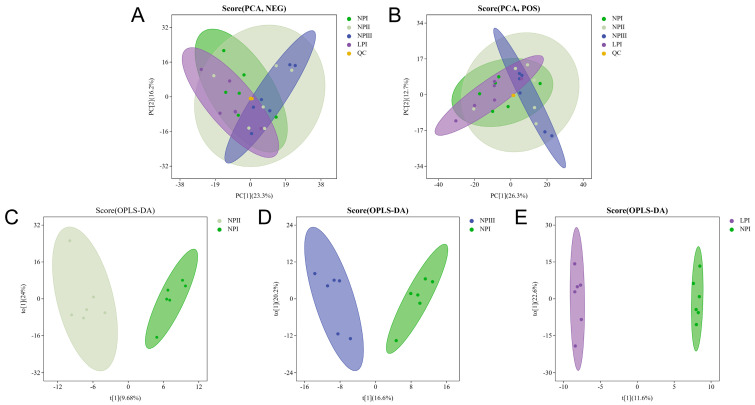
Multivariate analysis of fecal metabolomic profiles in growing beagles. (**A**,**B**) Principal component analysis (PCA) score plots of quality control samples in positive and negative ion modes, showing good analytical reproducibility and data stability. (**C**–**E**) Orthogonal partial least squares-discriminant analysis (OPLS-DA) score plots showing separation between groups: (**C**) NPII vs. NPI, (**D**) NPIII vs. NPI, and (**E**) LPI vs. NPI, indicating distinct fecal metabolic profiles among dietary treatments.

The PCA score plots showed that the quality control samples clustered tightly in both positive and negative ion modes, indicating good analytical reproducibility and data stability (Figure 3A,B). The experimental samples showed slight separation among groups, but the overall distribution suggested a relatively high similarity in fecal metabolite composition.

#### 3.5.2. Orthogonal Partial Least-Squares Discriminant Analysis (OPLS-DA)

OPLS-DA was further performed to evaluate metabolic differences between dietary treatments. Clear separation was observed in the pairwise comparisons of NPII vs. NPI, NPIII vs. NPI, and LPI vs. NPI (Figure 3C–E), indicating that arginine supplementation and a reduced dietary protein level induced distinct changes in fecal metabolic profiles.

#### 3.5.3. Differential Metabolites and Classification Statistics

**Figure 4:**
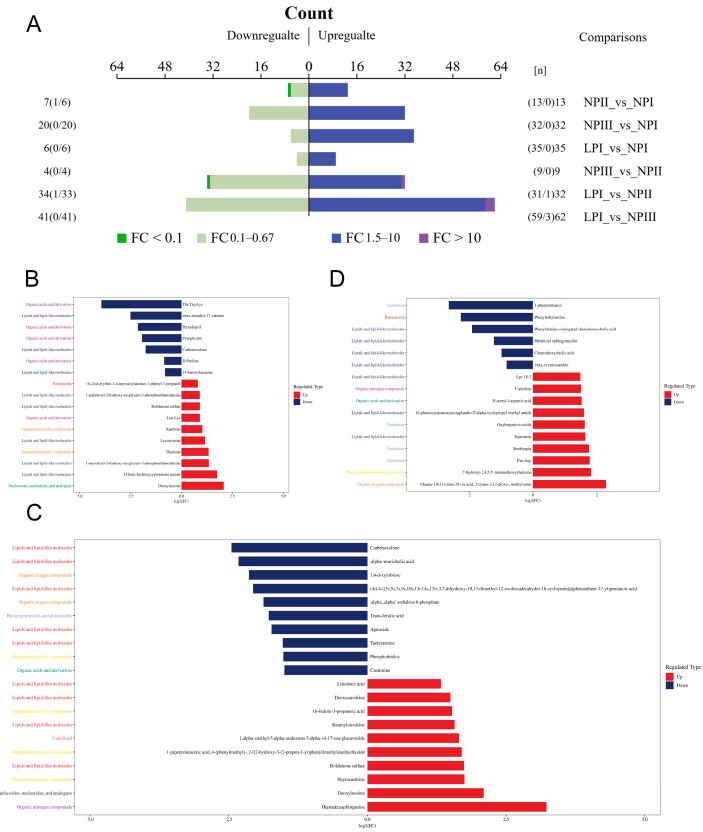
Differential metabolite analysis and chemical classification. (**A**) Number of significantly up-regulated and down-regulated metabolites identified in each comparison. (**B**–**D**) Chemical classification of differential metabolites for (**B**) NPII vs. NPI, (**C**) NPIII vs. NPI, and (**D**) LPI vs. NPI. Only these four representative groups (NPI, NPII, NPIII, and LPI) were included in the metabolomic analysis.

In total, 2135 metabolites were successfully identified across all samples. Compared with the NPI group, 13 up-regulated and 7 down-regulated metabolites were identified in the NPII group. In the NPIII group, 32 metabolites were up-regulated and 20 were down-regulated. In the LPI group, 35 metabolites were up-regulated and 6 were down-regulated (Figure 4A). Chemical classification analysis showed that the differential metabolites were mainly assigned to lipids and lipid-like molecules, organic acids and derivatives, and organoheterocyclic compounds (Figure 4B–D).

#### 3.5.4. KEGG Pathway Analysis

**Figure 5:**
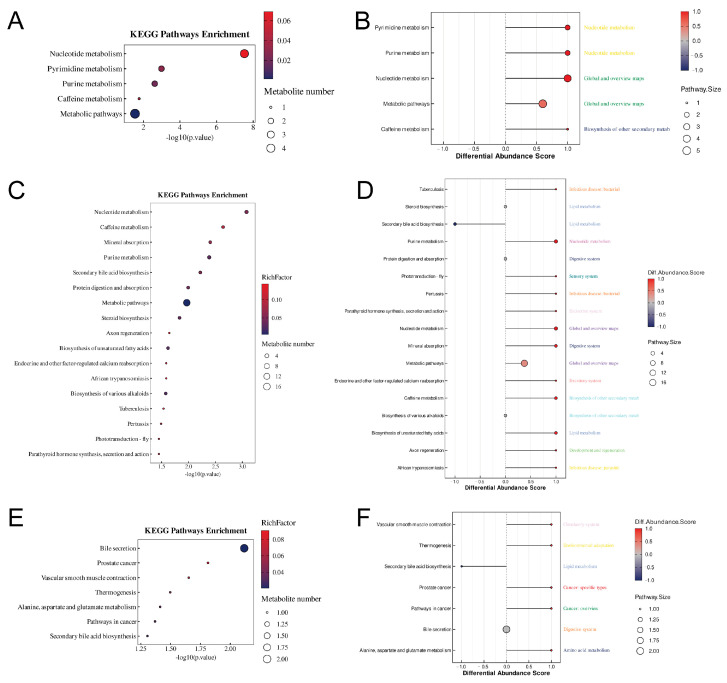
KEGG pathway enrichment analysis of differential metabolites. (**A**,**C**,**E**) Enriched metabolic pathways identified in pairwise comparisons: (**A**) NPII vs. NPI, (**C**) NPIII vs. NPI, and (**E**) LPI vs. NPI. (**B**,**D**,**F**) Differential abundance score plots of enriched KEGG pathways for the corresponding comparisons. Metabolomic analysis was performed only in the NPI, NPII, NPIII, and LPI groups.

Significantly enriched pathways were identified through pairwise comparisons and are shown in Figure 5A,C,E. Differential abundance score plots of the enriched KEGG pathways are shown in Figure 5B,D,F. In the comparison of NPII vs. NPI, the differential metabolites were mainly enriched in taurine and hypotaurine metabolism, arginine and proline metabolism, and D-amino acid metabolism. In the comparison of NPIII vs. NPI, enriched pathways included mineral absorption, secondary bile acid biosynthesis, protein digestion and absorption, and arginine and proline metabolism. In the comparison of LPI vs. NPI, the enriched pathways mainly included bile secretion, vascular smooth muscle contraction, and thermogenesis. These results suggest that arginine supplementation was primarily associated with changes in amino acid metabolism, particularly arginine and proline metabolism. At the higher supplementation level, arginine supplementation was also associated with bile acid-related metabolic pathways. In contrast, reducing the dietary crude protein level was mainly associated with changes in bile secretion, energy metabolism, and host metabolic regulation.

### 3.6. Correlation Analysis

**Figure 6:**
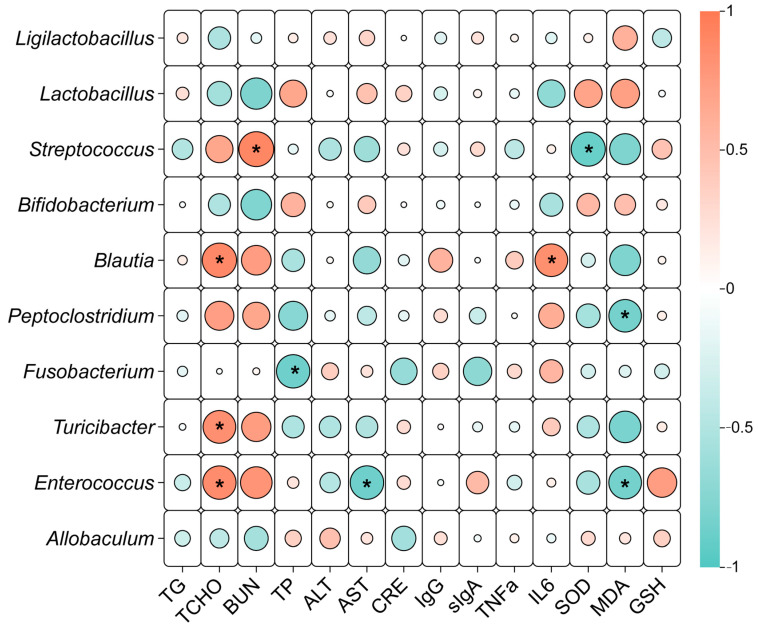
Heatmap of correlation between genus level and serum indicator. The color gradient represents the correlation coefficient (r), with red indicating positive correlations and blue indicating negative correlations. The size of each circle represents the absolute value of the correlation coefficient. * indicates a significant correlation (*p* < 0.05).

To further investigate the potential associations between differential bacterial taxa and host metabolic and inflammatory status, Spearman correlation analysis was performed between the major differential genera and serum biochemical parameters (Figure 6). The results revealed significant correlations between specific bacterial genera and serum indicators. *Streptococcus* exhibited a significant positive correlation with BUN and a significant negative correlation with SOD (*p* < 0.05). *Blautia* showed significant positive correlations with TCHO and IL-6 (*p* < 0.05). *Fusobacterium* was significantly negatively correlated with TP (*p* < 0.05). *Turicibacter* displayed a significant positive correlation with TCHO (*p* < 0.05). In addition, *Enterococcus* was positively correlated with TCHO and negatively correlated with AST and MDA (*p* < 0.05). These findings indicate that differentially abundant bacterial genera exhibit distinct correlation patterns with host metabolic parameters and inflammation-related indicators.

## 4. Discussion

This study evaluated the effects of dietary protein and arginine levels in growing beagles. Although growth performance was not significantly affected, slightly lower average daily gain in low-protein diets with higher arginine suggests a potential negative effect of excessive arginine under protein-restricted conditions. Nutrient digestibility remained unchanged across treatments. Reduced urinary nitrogen excretion in low-protein diets reflected lower nitrogen intake, while increased biological value indicated improved nitrogen utilization. These findings suggest improved apparent nitrogen utilization efficiency under the low-protein formulation, although absolute nitrogen retention was not significantly increased. As a key component of the urea cycle, excess arginine may increase urea synthesis and nitrogen excretion [16]. Alternatively, imbalanced amino acid supply may reduce protein synthesis efficiency and promote amino acid catabolism, contributing to the observed changes in nitrogen metabolism [17].

TG are derived from dietary fat or hepatic synthesis and are closely associated with energy metabolism [18]. In the present study, TG levels tended to be higher in the LPI group, although these differences were not statistically significant. Crude fat digestibility did not differ among groups, suggesting that the numerical increase was unlikely due to enhanced lipid absorption. Instead, this numerical increase may suggest a potential alteration in lipid metabolism under low-protein conditions, although this interpretation requires further validation. Similar findings have been reported in mice [19]. A decreasing trend in TG levels was observed across the LPI, LPII, and LPIII groups, suggesting a potential modulatory role of arginine supplementation that requires further investigation. These observations are consistent with previous studies in broilers, where dietary L-arginine reduced plasma TG levels, potentially via downregulation of fatty acid synthase and enhancement of fatty acid β-oxidation-related enzymes [20]. T-CHO is a key indicator of lipid metabolism [21]. T-CHO is derived partly from dietary intake and largely from hepatic synthesis, and plays essential roles in membrane structure, steroid hormone production, and bile acid synthesis [22]. In this study, serum T-CHO decreased with reduced dietary protein levels, consistent with previous findings [23]. Additionally, at the same protein level, T-CHO showed a decreasing trend with increasing arginine supplementation, which agrees with earlier reports [24]. These findings suggest that both dietary protein and specific amino acids play important roles in regulating lipid metabolism and body composition, independent of total energy intake [25]. However, further studies incorporating body composition measurements are needed to clarify whether arginine supplementation affects lean mass or adiposity in growing dogs.

Serum CRE is a commonly used indicator of renal function [26]. Creatinine is generated from creatine metabolism in muscle and is released into the bloodstream before being excreted by the kidneys [27,28]. Impaired renal function is typically associated with elevated serum CRE concentrations [29]. In the present study, serum CRE levels were reduced in some treatment groups. This decrease may be associated with altered protein metabolism or reduced muscle catabolism following arginine supplementation, although the underlying mechanism remains unclear [30]. Overall, these findings suggest that arginine supplementation did not adversely affect renal function in growing dogs. ALT, AST, and TP are commonly used indicators of liver function, nutritional status, and immune condition [31], whereas SOD, MDA, and GSH are widely used to evaluate oxidative stress status [32]. In this study, no significant differences were observed among groups for these parameters, indicating that dietary protein and arginine levels did not negatively affect liver function or oxidative status in dogs.

TNF-α and IL-6 are important pro-inflammatory cytokines that play a central role in immune defense and inflammatory responses [33]. In the present study, TNF-α was not significantly affected by the main effects of dietary protein or arginine; however, a significant CP × Arg interaction was observed, indicating that the regulatory effect of arginine depended on protein level. Notably, TNF-α levels showed a decreasing trend with increasing arginine supplementation under both protein levels, suggesting a potential anti-inflammatory role of arginine. For IL-6, dietary protein level had a significant effect, and a significant interaction between CP and Arg was also detected. Under the LP condition, arginine supplementation significantly reduced IL-6 levels, whereas no significant differences were observed among treatments under the NP condition. These results indicate that arginine exerts a more pronounced anti-inflammatory effect under protein-restricted conditions. Taken together, the present findings suggest that the effects of arginine on inflammatory cytokines are dependent on dietary protein level, with a clearer anti-inflammatory response observed under low-protein conditions. This may be related to differences in amino acid availability and nitrogen metabolism, which could influence immune signaling pathways [34].

In terms of gut microbiota, alpha diversity analysis showed that the NPIII group exhibited significantly higher Chao1 and Shannon indices, suggesting that high-level arginine supplementation increased microbial richness under normal protein conditions. Arginine may serve as an additional nitrogen source, supporting microbial growth and diversity [35]. However, no significant differences were observed in the Simpson index, indicating that community evenness remained stable. Beta diversity analysis showed no clear separation among groups, suggesting that dietary treatments primarily affected microbial abundance rather than overall community structure. Nevertheless, the detection of differentially abundant taxa indicates that dietary protein levels and arginine supplementation may selectively regulate specific bacterial populations, resulting in compositional shifts within a relatively stable microbial ecosystem.

A key finding was the enrichment of *Lactobacillaceae* and *Lactobacillus* in low-protein diets supplemented with arginine. Under low-protein conditions, reduced undigested protein reaching the hindgut limits the proliferation of proteolytic bacteria and decreases harmful metabolites such as ammonia and amines [36]. In this context, dietary arginine supplementation may influence microbial nitrogen metabolism [35]. *Lactobacillus* species possess the arginine deiminase pathway, which is involved in arginine utilization and energy conservation under nutrient-limited conditions [4,37]. Therefore, the enrichment of Lactobacillus observed in the present study may reflect an adaptive microbial response to altered dietary arginine availability. In contrast, under normal protein conditions, abundant amino acids dilute the specific effect of arginine supplementation, while increased protein fermentation may inhibit beneficial bacteria. Similar findings have been reported in studies where dietary protein levels modulated gut microbial composition [38,39].

The enrichment of *Clostridia*, *Peptoclostridium*, and *Fusobacterium* in the NPII group suggests enhanced protein and amino acid fermentation under normal protein conditions. *Clostridia* are well known for Stickland amino acid fermentation, in which amino acids, including branched-chain amino acids, can be used as electron donors or acceptors to support anaerobic energy metabolism [40]. Intestinal bacteria-derived amino acid metabolites may influence host health by regulating microbial composition, microbial metabolism, host immunity, and cellular function [41]. In addition, nitrogenous compounds derived from luminal proteins and amino acids can be metabolized by the gut microbiota into products such as ammonia, short-chain fatty acids, and branched-chain fatty acids [42]. Canine-derived *Fusobacterium varium* showed strong adhesion to healthy beagle colonic mucosa and was proposed to have potential probiotic properties, although further safety and efficacy studies are still required [43]. The NPIII group showed enrichment of *Bacteroidota* and *Prevotellaceae*, taxa associated with carbohydrate degradation and metabolic flexibility [44]. Their increase may be associated with altered nitrogen availability following arginine supplementation, potentially contributing to changes in microbial diversity and metabolic activity. Previous studies have linked *Prevotella* abundance with enhanced microbial diversity and metabolic benefits [45]. Overall, the microbial response to arginine supplementation appeared to vary with dietary protein level. Under low-protein conditions, arginine supplementation was associated with increased abundance of *Lactobacillus*. In this study, non-targeted fecal metabolomics was performed in four selected representative groups, including NPI, NPII, NPIII, and LPI, to further explore the metabolic responses associated with arginine supplementation and dietary crude protein reduction. Compared with the NPI group, the NPII group showed alterations in metabolites related to taurine and hypotaurine metabolism and D-amino acid metabolism, including hypotaurine and D-proline. The reduced fecal hypotaurine level may indicate changes in taurine and hypotaurine metabolism. Taurine-related metabolism has been reported to be involved in antioxidant defense, inflammatory regulation, and intestinal function [46]. Therefore, this pathway may partly contribute to the metabolic response induced by moderate arginine supplementation.

Compared with the NPI group, the NPIII group showed broader metabolic alterations, involving mineral absorption, protein digestion and absorption, arginine and proline metabolism, and secondary bile acid biosynthesis. Because fecal metabolites represent the combined effects of host metabolism, microbial fermentation, and unabsorbed dietary components, these changes should be interpreted as alterations in intestinal metabolic profiles rather than direct indicators of systemic host metabolism. Several differential metabolites were identified, including up-regulated calcitriol, DL-tryptophan, and 5-aminovaleric acid, and down-regulated ursocholic acid, α-muricholic acid, and creatinine. Calcitriol, the active form of vitamin D_3_, plays an important role in calcium and phosphorus homeostasis and intestinal calcium absorption [47]. The increased fecal calcitriol level was consistent with the enrichment of the mineral absorption pathway, suggesting that high-level arginine supplementation under the normal-protein diet may be associated with altered mineral-related metabolic processes. Previous studies have shown that arginine supplementation may enhance calcium utilization [48,49], and the increased calcitriol level observed here may be associated with this effect [50].

In the LPI group, metabolic differences compared with the NPI group were mainly related to lipid metabolism, including bile secretion and thermogenesis. Increased fecal carnitine and decreased chenodeoxycholic acid suggested that reducing dietary crude protein may influence fatty acid utilization and bile acid metabolism [51], consistent with previous findings [52]. The increased level of 16-hydroxyhexadecanoic acid may also suggest altered lipid metabolic activity, although its specific physiological relevance in growing dogs remains uncertain [53]. Overall, the fecal metabolomic results suggest that arginine supplementation under the normal-protein diet mainly affected amino acid-related metabolism, mineral absorption, and bile acid-related pathways, whereas crude protein reduction primarily influenced lipid metabolism, bile secretion, and energy-related pathways. However, because metabolomic analysis was conducted only in selected representative groups rather than all six dietary treatments, these findings should be interpreted as exploratory. Further targeted metabolomic analyses and validation in all treatment groups are needed to clarify the metabolic mechanisms underlying the effects of dietary protein level and arginine supplementation in growing beagles.

One limitation of this study is that fecal metabolomic analysis was conducted only in four representative groups (NPI, NPII, NPIII, and LPI), excluding LPII and LPIII. Thus, although these data revealed metabolic changes associated with arginine supplementation under normal-protein conditions and low-protein feeding alone, they cannot statistically determine the interaction between dietary protein level and arginine supplementation and should therefore be interpreted as exploratory. Moreover, despite essential amino acid supplementation in low-protein diets, differences in non-essential amino acids, total amino acid supply, and ingredient composition remained between low- and normal-protein diets, which may have contributed to fecal metabolic alterations. Future studies integrating complete factorial metabolomic designs and more precisely balanced diets are warranted to distinguish the effects of protein level from amino acid composition and to clarify LP × Arg metabolic interactions. In addition, the use of corn protein powder as the primary protein source may limit the direct application of these findings to commercial diets containing higher proportions of animal-derived proteins. Future studies using more representative commercial formulations are warranted to further confirm these results.

## 5. Conclusions

In conclusion, reducing dietary crude protein from 22% to 18% did not significantly affect growth performance or apparent nutrient digestibility in growing beagles. The low-protein diet decreased nitrogen intake and urinary nitrogen excretion while increasing biological value, suggesting improved nitrogen utilization efficiency under nutritionally adequate dietary conditions. Arginine supplementation did not further improve growth performance or nutrient digestibility, but exerted protein-level-dependent effects on serum biochemical parameters, inflammatory cytokines, gut microbiota composition, and fecal metabolic profiles. Microbiota analysis and fecal metabolomic analysis conducted in four selected dietary groups suggested that arginine supplementation under normal-protein conditions was mainly associated with changes in amino acid metabolism, mineral absorption, bile acid-related pathways, and specific microbial taxa, whereas dietary protein reduction was more closely related to alterations in lipid metabolism, bile secretion, and energy-related pathways. Under the conditions of the present study, a normal-protein diet supplemented with moderate arginine (1.10%) showed favorable physiological responses compared with high-dose arginine (1.35%) supplementation; however, because metabolomic analyses were not performed in all dietary groups, further studies are required to determine the optimal arginine level.

## Figures and Tables

**Table 1:** Ingredient composition and Nutritional levels of the experimental diets (dry matter basis).

Items	NPI	NPII	NPIII	LPI	LPII	LPIII
Ingredients (%)						
Puffed corn	22.4	22.4	22.4	32.4	32.4	32.4
Cooked rice flour	12	12	12	12	12	12
Cooked wheat flour	7	7	7	7	7	7
Cooked tapioca flour	4.8	4.534	4.268	4.8	4.534	4.268
Corn protein powder	26	26	26	16	16	16
Meat and bone meal	4	4	4	4	4	4
Alfalfa grass powder	2	2	2	2	2	2
Dicalcium phosphate	2	2	2	2	2	2
Whey powder	7.8	7.8	7.8	7.8	7.8	7.8
Chicken fat	11	11	11	11	11	11
(Premix ^1^)	1	1	1	1	1	1
L-Arginine	0	0.266	0.532	0	0.266	0.532
Total	100	100	100	100	100	100
Nutrient (%)						
DM	94.46	94.27	94.18	94.22	94.03	94.08
Crude protein	23.91	23.46	23.72	18.96	19.18	19.40
Crude fat	14.02	14.17	14.75	15.71	15.88	15.22
Crude ash	4.25	4.35	4.29	4.19	4.14	4.12
Ca	1.13	1.13	1.05	1.11	1.05	1.11
P	0.79	0.81	0.80	0.78	0.77	0.79
ME (MJ/kg *)	18.11	18.05	18.21	18.18	18.24	18.11
GE (MJ/kg *)	21.13	21.12	21.28	21.19	21.25	21.12
Arg (total)	0.85	1.10	1.35	0.85	1.08	1.33

*^,1^, Metabolizable energy (ME) and gross energy (GE) were calculated values, whereas all other nutrient levels were analyzed directly.

**Table 2:** Effects of dietary crude protein level and arginine supplementation on growth performance in growing beagles (*n* = 10).

Items	22% CP			18% CP			*p*-Value of Two-Way ANOVA		
	0% Arg	0.266% Arg	0.532% Arg	0% Arg	0.266% Arg	0.532% Arg	CP	Arg	CP × Arg Interaction
Initial BW, kg	5.02 ± 1.18	5.07 ± 0.75	4.97 ± 1.12	4.99 ± 0.83	5.12 ± 1.09	5.22 ± 0.96	0.739	0.946	0.898
15 d BW, kg	5.61 ± 1.30	5.52 ± 0.92	5.40 ± 1.19	5.44 ± 1.18	5.58 ± 1.36	5.52 ± 0.95	0.996	0.971	0.918
30 d BW, kg	6.35 ± 1.40	6.51 ± 0.74	6.13 ± 1.56	6.36 ± 1.37	6.42 ± 1.58	6.44 ± 1.12	0.831	0.909	0.887
Final BW, kg	6.80 ± 1.51	6.63 ± 1.37	6.67 ± 1.46	6.68 ± 1.29	6.48 ± 1.29	6.72 ± 1.12	0.844	0.913	0.969
ADFI, g/d	224.18 ± 47.56	236.74 ± 25.74	235.28 ± 50.77	235.85 ± 36.06	233.33 ± 46.86	243.27 ± 42.48	0.631	0.790	0.853
ADG, g	39.44 ± 9.68	35.56 ± 16.90	37.67 ± 11.07	37.56 ± 12.07	33.83 ± 8.79	33.33 ± 12.25	0.405	0.586	0.930

Note: No significant differences were detected among treatments.

**Table 3:** Effects of dietary crude protein level and arginine supplementation on apparent nutrient digestibility and nitrogen metabolism in growing beagles (*n* = 10).

Items	22% CP			18% CP			*p*-Value of Two-Way ANOVA		
	0% Arg	0.266% Arg	0.532% Arg	0% Arg	0.266% Arg	0.532% Arg	CP	Arg	CP × Arg Interaction
DM,%	83.15 ± 3.54	83.49 ± 2.34	83.69 ± 4.46	85.61 ± 3.22	85.43 ± 2.45	84.65 ± 1.62	0.032	0.955	0.745
CP,%	74.81 ± 7.61	74.65 ± 4.47	78.17 ± 6.49	74.57 ± 6.86	74.03 ± 6.57	71.61 ± 4.44	0.150	0.964	0.251
EE,%	94.12 ± 2.08	94.60 ± 2.26	95.22 ± 2.39	96.15 ± 2.20	95.48 ± 1.37	94.69 ± 1.34	0.137	0.961	0.143
Ash,%	37.14 ± 7.18	36.43 ± 6.24	40.10 ± 8.83	39.74 ± 10.56	37.30 ± 7.53	36.85 ± 7.82	0.972	0.785	0.524
Ca,%	42.46 ± 10.01	44.69 ± 8.09	39.89 ± 10.74	41.49 ± 11.88	36.61 ± 9.00	42.04 ± 7.81	0.367	0.905	0.252
P,%	45.65 ± 5.92	47.60 ± 8.16	44.64 ± 11.23	44.65 ± 12.97	40.40 ± 7.12	44.21 ± 8.08	0.240	0.926	0.459
NI, g/d	9.03 ± 1.13 ^a^	8.65 ± 1.29 ^a^	8.88 ± 0.89 ^a^	6.70 ± 0.68 ^b^	6.98 ± 0.65 ^b^	7.18 ± 0.85 ^b^	<0.001	0.759	0.473
FN, g/d	2.40 ± 0.78	2.16 ± 0.39	2.58 ± 1.06	2.22 ± 1.43	1.79 ± 0.38	2.04 ± 0.44	0.105	0.382	0.797
UN, g/d	3.34 ± 1.14 ^a^	3.63 ± 0.63 ^a^	3.78 ± 0.97 ^a^	2.00 ± 0.93 ^b^	2.41 ± 0.69 ^b^	2.71 ± 0.33 ^b^	<0.001	0.094	0.884
UR, g/d	3.30 ± 0.93	2.85 ± 0.95	2.52 ± 1.03	2.48 ± 0.92	2.78 ± 0.69	2.43 ± 0.58	0.156	0.280	0.314
NPU,%	36.41 ± 9.32	32.06 ± 7.73	30.55 ± 6.86	34.28 ± 9.92	39.52 ± 8.02	33.40 ± 5.87	0.208	0.290	0.197
BV,%	46.92 ± 7.37	46.80 ± 6.75	41.82 ± 6.86 ^b^	50.79 ± 5.99	48.30 ± 3.10	48.82 ± 4.64 ^a^	0.021	0.227	0.431

Note: Within the same Arg level, means with different lowercase superscripts differ significantly between CP levels (*p* < 0.05), as determined by Duncan’s multiple range test.

**Table 4:** Effects of dietary crude protein level and arginine supplementation on serum biochemical parameters in growing beagles (*n* = 10).

Items	22% CP			18% CP			*p*-Value of Two-Way ANOVA		
	0% Arg	0.266% Arg	0.532% Arg	0% Arg	0.266% Arg	0.532% Arg	CP	Arg	CP × Arg Interaction
TG, mmol/L	0.52 ± 0.08	0.54 ± 0.09	0.58 ± 0.12	0.68 ± 0.12	0.57 ± 0.16	0.51 ± 0.16	0.338	0.464	0.040
T-CHO, mmol/L	3.47 ± 0.69 ^Aa^	2.42 ± 0.45 ^Ba^	2.49 ± 0.18 ^Ba^	2.47 ± 0.47 ^Ab^	1.98 ± 0.21 ^Bb^	1.65 ± 0.26 ^Bb^	<0.001	<0.001	0.167
BUN, mmol/L	3.94 ± 0.69 ^Aa^	2.89 ± 0.64 ^B^	2.83 ± 0.84 ^Ba^	2.24 ± 0.63 ^Ab^	2.78 ± 0.51 ^A^	1.68 ± 0.43 ^Bb^	<0.001	<0.001	0.004
TP, g/L	66.05 ± 5.55	58.21 ± 8.23	65.28 ± 7.13	64.71 ± 7.07	66.27 ± 9.52	68.01 ± 6.79	0.152	0.242	0.216
ALT, U/L	4.69 ± 0.99	4.99 ± 0.46	5.68 ± 1.27	5.01 ± 1.46	4.88 ± 1.22	5.00 ± 0.75	0.634	0.447	0.476
AST, U/L	2.62 ± 2.06	4.04 ± 2.70	3.22 ± 1.19	3.84 ± 1.41	3.90 ± 2.10	4.20 ± 0.90	0.233	0.560	0.577
CRE, µmol/L	75.17 ± 4.17 ^A^	68.88 ± 6.20 ^Bb^	61.73 ± 3.11 ^Cb^	77.47 ± 3.64 ^A^	76.89 ± 3.17 ^Aa^	70.99 ± 3.59 ^Ba^	<0.001	<0.001	0.048

Note: Within the same CP level, means with different uppercase superscripts differ significantly among Arg levels (*p* < 0.05). Within the same Arg level, means with different lowercase superscripts differ significantly between CP levels (*p* < 0.05), as determined by Duncan’s multiple range test.

**Table 5:** Effects of dietary crude protein level and arginine supplementation on serum immune and antioxidant parameters in growing beagles (*n* = 10).

Items	22% CP			18% CP			*p*-Value of Two-Way ANOVA		
	0% Arg	0.266% Arg	0.532% Arg	0% Arg	0.266% Arg	0.532% Arg	CP	Arg	CP × Arg Interaction
IgG, µg/mL	255.17 ± 56.40 ^B^	260.29 ± 48.05 ^AB^	327.67 ± 76.23 ^A^	276.95 ± 56.76 ^A^	214.10 ± 28.43 ^B^	233.50 ± 61.41 ^AB^	0.029	0.132	0.034
sIgA, µg/mL	1.37 ± 0.50	1.25 ± 0.58	1.36 ± 0.33	1.32 ± 0.41	1.36 ± 0.24	1.32 ± 0.43	0.981	0.966	0.868
TNF-α, pg/mL	69.24 ± 14.51	54.70 ± 5.64	50.02 ± 8.25	61.07 ± 7.69	50.28 ± 11.18	48.84 ± 12.76	0.164	0.266	0.001
IL-6, pg/mL	343.19 ± 69.18	307.97 ± 55.42	287.44 ± 55.19	282.23 ± 28.61 ^A^	232.06 ± 65.00 ^AB^	210.30 ± 54.16 ^B^	<0.001	0.802	0.025
SOD, U/mL	3.26 ± 1.12	3.39 ± 1.01	3.95 ± 1.10	4.37 ± 1.27	3.61 ± 1.11	3.86 ± 1.22	0.221	0.582	0.318
MDA, nmoL/mL	0.44 ± 0.09	0.53 ± 0.22	0.56 ± 0.11	0.61 ± 0.11	0.61 ± 0.10	0.61 ± 0.14	0.011	0.503	0.422
GSH, µmoL/mL	0.29 ± 0.07	0.26 ± 0.04	0.27 ± 0.03	0.25 ± 0.03	0.26 ± 0.04	0.28 ± 0.05	0.578	0.597	0.317

Note: Within the same CP level, means with different uppercase superscripts differ significantly among Arg levels (*p* < 0.05), as determined by Duncan’s multiple range test.

## Data Availability

The data is submitted to the NCBI database with the project number PRJNA1468964.

## Supplementary Materials

The following supporting information can be downloaded at https://www.mdpi.com/article/10.3390/ani16152385/s1, Table S1: Composition of the diet (dry matter basis); Table S2: Amino acid composition of the main raw materials in the diet.

## Abbreviations

The following abbreviations are used in this manuscript:

Abbreviation	Full name
FN	fecal nitrogen
BW	body weight
NI	Nitrogen intake
BV	biological value
NR	nitrogen retention
CP	crude protein
TP	total protein
CRE	creatinine
TG	triglycerides
NEU	neutrophil
LYM	lymphocyte
EOS	eosinophil
GSH	glutathione
MDA	malondialdehyde
IgG	immunoglobulin G
IL-6	interleukin-6
ADFI	average daily feed intake
ADG	average daily gain
UN	urinary nitrogen excretion
NPU	net protein utilization
ALT	alanine aminotransferase
AST	aspartate aminotransferase
BUN	Blood urea nitrogen
T-CHO	total cholesterol
HDL-C	high-density lipoprotein cholesterol
LDL-C	low-density lipoprotein cholesterol
WBC	white blood cell
SOD	superoxide dismutase
sIgA	secretory immunoglobulin A
TNF-α	tumor necrosis factor-α
GLM	general linear model
CDCA	Chenodeoxycholic Acid
