# Supplementary material for: Dietary Arginine Supplementation Modulates Nitrogen Utilization, Serum Biochemistry, and Gut Microbiota in Growing Beagles Under Low- and Normal-Protein Diets
Source: Animals (Basel). 2026 Aug 3;16(15):2385. doi: 10.3390/ani16152385 (PMC13466215; doi:10.3390/ani16152385)
Supplement: Supplementary file 1 [file animals-16-02385-s001.zip › animals-4430593-supplementary.pdf]

Table S1. ***Composition of the diet(dry matter basis)***

Ingredients,%	NP	LP
Puffed corn	22.4	30.7
Cooked rice flour	12	12
Cooked wheat flour	7	7
Cooked tapioca flour	4.8	4.8
Corn protein powder	26	16
Meat and bone meal	4	4
Alfalfa grass powder	2	2
Dicalcium phosphate	2	2
Whey powder	7.8	7.8
Chicken fat	11	11
Premix[1]	1	1
Arg		0.12
Ile	0.1	0.36
Met		0.12
Phe		0.28
Tyr		0.22
Thr		0.14
Ser	0.1	0.1
Val		0.2
Lys	0.1	0.16
Total	100	100

Notes. [1]The premix provided the following per kg of diets: VA 8 000 IU, VD3 2 000 IU, VE 100 IU, VK_3 60 mg, VB_1 6 mg, VB_2 4 mg, VB_6 5 mg, VB_{12} 0.3 mg, VC 400 mg, nicotinic acid 50 mg, calcium pantothenate 75 mg, folic acid 1.5 mg, Biotin 1 mg, choline 1400 mg, Fe 150 mg, Cu 12 mg, Zn 200 mg, Mn 40 mg, I 2 mg, Mg 1.5 mg, Se 0.3 mg.

Table S2 ***Amino acid composition of the main raw materials in the diet***

Items	Corn protein powder	Meat and bone meal	Whey powder	Puffed corn	Cooked rice flour	Cooked wheat flour	Alfalfa grass powder
Asp	3.41	4.94	1.23	0.58	0.76	0.59	1.8
Thr	1.94	2.39	0.80	0.33	0.30	0.33	0.75
Ser	2.82	2.52	0.58	0.42	0.42	0.50	0.75
Glu	12.83	8.16	2.08	1.68	1.62	3.35	2.28
Gly	1.69	5.52	0.25	0.36	0.37	0.48	0.90
Ala	5.29	3.89	0.57	0.67	0.49	0.44	0.94
Cys	0.50	0.34	0.10	0.06	0.06	0.11	0.04
Val	2.75	2.92	0.72	0.45	0.52	0.54	0.99
Met	1.54	1.26	0.19	0.15	0.13	0.15	0.08
Ile	2.43	2.42	0.68	0.32	0.36	0.43	0.72
Leu	9.48	4.31	1.16	1.09	0.73	0.79	1.29
Tyr	2.81	1.74	0.28	0.24	0.24	0.35	0.53
Phe	3.58	2.55	0.38	0.44	0.47	0.55	0.90
Lys	0.93	4.23	1.08	0.28	0.31	0.34	1.12
His	1.18	1.37	0.21	0.26	0.20	0.27	0.4
Arg	1.76	4.09	0.28	0.40	0.64	0.55	0.88
Pro	5.64	3.47	0.67	0.78	0.40	1.13	0.90
TAA	60.59	56.11	11.25	8.50	8.02	10.89	15.26

Notes. Data are expressed as percentages (%).This table contains the composition of 17 amino acids in some raw materials in the diet. TAA: Total Amino Acids.
